# Performance outcomes from a DMEK peeling and preparation wet lab

**DOI:** 10.1136/bmjophth-2023-001540

**Published:** 2024-01-25

**Authors:** Mohit Parekh, Alexander George Wallace, Matteo Airaldi, Alessandro Ruzza, Stefano Ferrari, Vito Romano, Sajjad Ahmad

**Affiliations:** 1 Schepens Eye Research Institute of Massachusetts Eye and Ear, Boston, MA, USA; 2 Department of Ophthalmology, Harvard Medical School, Boston, MA, USA; 3 University of Liverpool, Liverpool, UK; 4 Department of Medical and Surgical Specialties, Radiological Sciences, and Public Health, University of Brescia, Brescia, Italy; 5 Fondazione Banca degli Occi del Veneto, Mestre, Venice, Italy; 6 Moorfields Eye Hospital NHS Foundation Trust, London, UK; 7 Institute of Ophthalmology, University College London, London, UK

**Keywords:** Cornea, Eye (Tissue) Banking

## Abstract

**Objective:**

To evaluate the Descemet membrane endothelial keratoplasty (DMEK) preparation performance of trainee surgeons in an ex vivo human donor cornea DMEK wet lab simulation setting.

**Methods:**

Human donor corneoscleral rims unsuitable for transplantation were obtained from Moorfields Lions Eye Bank. At the wet lab, graft stripping was performed by scoring the peripheral endothelium. The trypan blue positive cells (TBPC) and cell density (cells/mm^2^—reticule count) were counted manually before and after stripping. The procedural time, peripheral and central tears and complete peel-off were also recorded and analysed.

**Results:**

Eight trainee surgeons attended the wet lab each attempting three DMEKs. Between the first and last attempts a significant decrease was seen in the procedural time (17.6 min vs 10.6 min (p<0.05)) and the TBPC % (12.9% vs 3.8% (p<0.05)). The percentage of tears peripherally and centrally also reduced between the first and the last trials (50% vs 13% (p=0.2226) and 38% vs 0% (p=0.1327)). A significant correlation was found between longer peeling times and higher TBPC % (p<0.001) with a 0.7% endothelial mortality increase for each additional minute required to complete the peel.

**Conclusions:**

DMEK wet labs provide a controlled risk-free learning opportunity for trainee surgeons to improve confidence and competence. Wet labs improve the success rate of DMEK graft preparation as well as flatten the learning curve. This emphasises the importance of continued support for the expansion of this valuable learning resource, promoting wider uptake of DMEK surgery.

**WHAT IS ALREADY KNOWN ON THIS TOPIC**

- It is subjectively known that Descemet membrane endothelial keratoplasty (DMEK) wet labs facilitate enhancing surgical skills in ex vivo settings.
- The learning curve and skill set development after attending a DMEK wet lab has never been objectively quantified.

**WHAT THIS STUDY ADDS**

- We have quantified the main outcome measures like endothelial cell loss, mortality, time required to prepare a DMEK graft and the amount of tears after a DMEK wet lab, which has never been shown earlier.

**HOW THIS STUDY MIGHT AFFECT RESEARCH, PRACTICE OR POLICY**

- We show that DMEK wet labs are an essential learning tool for young surgeons to improve confidence and competence.
- Attending wet labs can improve the success rate of DMEK graft preparation and reduce the learning curve and tissue wastage.
- Practising in a risk-free environment further educates more surgeons to take up DMEK.

## Introduction

In recent decades, Descemet membrane endothelial keratoplasty (DMEK) has emerged as the preferential corneal transplant treatment for endothelial failure. DMEK has surpassed penetrating keratoplasty (PK) and Descemet stripping automated endothelial keratoplasty (DSAEK) due to minimal endothelial cell loss (ECL), rapid visual recovery, improved visual outcomes and lower rejection rate.^1–5^ Nonetheless, the adoption of DMEK among ophthalmic surgeons has been gradual due to the steep learning curve, as the thin DMEK graft is difficult to handle during preparation, loading and delivery.^6^ Less experienced surgeons might encounter more intraoperative complications and ECL. This, in turn, could lead to primary or early graft failure and restrict long-term survival.^7^ The aforementioned factors emphasise the importance of proficient DMEK surgical training. Wet labs have proven to be an effective method to cultivate surgical skills.^8–11^ Trainee surgeons practise the technical aspects of DMEK surgery in a controlled risk-free setting to develop confidence and improve surgical performance.^12^

Wet labs use ex vivo human donor corneoscleral rims not suitable for transplantation. Although expensive, it allows the complete DMEK procedure from graft preparation to delivery to be practised in a realistic environment.^12^ The human donor cornea is mounted on an artificial anterior chamber to create an authentic surgical set-up. Additionally, the graft is handled using the same instruments used during the surgery. Non-human models have also been used for DMEK training including animal tissues (pig eyes), vegetable matter (onion model) and synthetic material (artificial eye (AE)).^13 14^ While less expensive and more readily available, animal tissue and vegetable matter differ from humans in terms of consistency and size. AEs accurately resemble real human corneas though they are expensive and lack both a pupil and posterior segment, making them an inconvenient model for DMEK teaching.^12^

This paper aims to report the performance outcomes of trainee surgeons in an ex vivo human cornea DMEK graft preparation wet lab assessing the peeling and preparation time, ECL, graft tears and complete graft peels. This will provide a comprehensive understanding of the significance of wet labs in enhancing the training and adoption of DMEK surgery.

## Materials and methods

### Ethical statement and donor characteristics

Human donor corneal tissues were obtained from Moorfields Lions Eye Bank, London, UK, with written consent from the donor’s next of kin to be used for transplantation and, if unsuitable, for research purposes aiming at improving the process and training technicians and surgeons.

### Tissue collection and preservation

All the tissues were excised from the cadavers directly and preserved in the organ culture (OC) media at 37°C. The OC media was composed of a basal media (Mimimum essential medium-Earle) supplemented with 2% newborn calf serum, 25 mM HEPES buffer, 26 mM sodium bicarbonate, 1 mM pyruvate, 2 mM glutamine, 250 ng/mL amphotericin B, 100 IU/mL penicillin G and 100 mg/mL streptomycin. Following the OC step, the tissues were stored at 31°C for up to 4 days in the transport media which was supplemented with 6% dextran T-500 in the OC media.^15^

### Tissue evaluation

**Figure 1:**
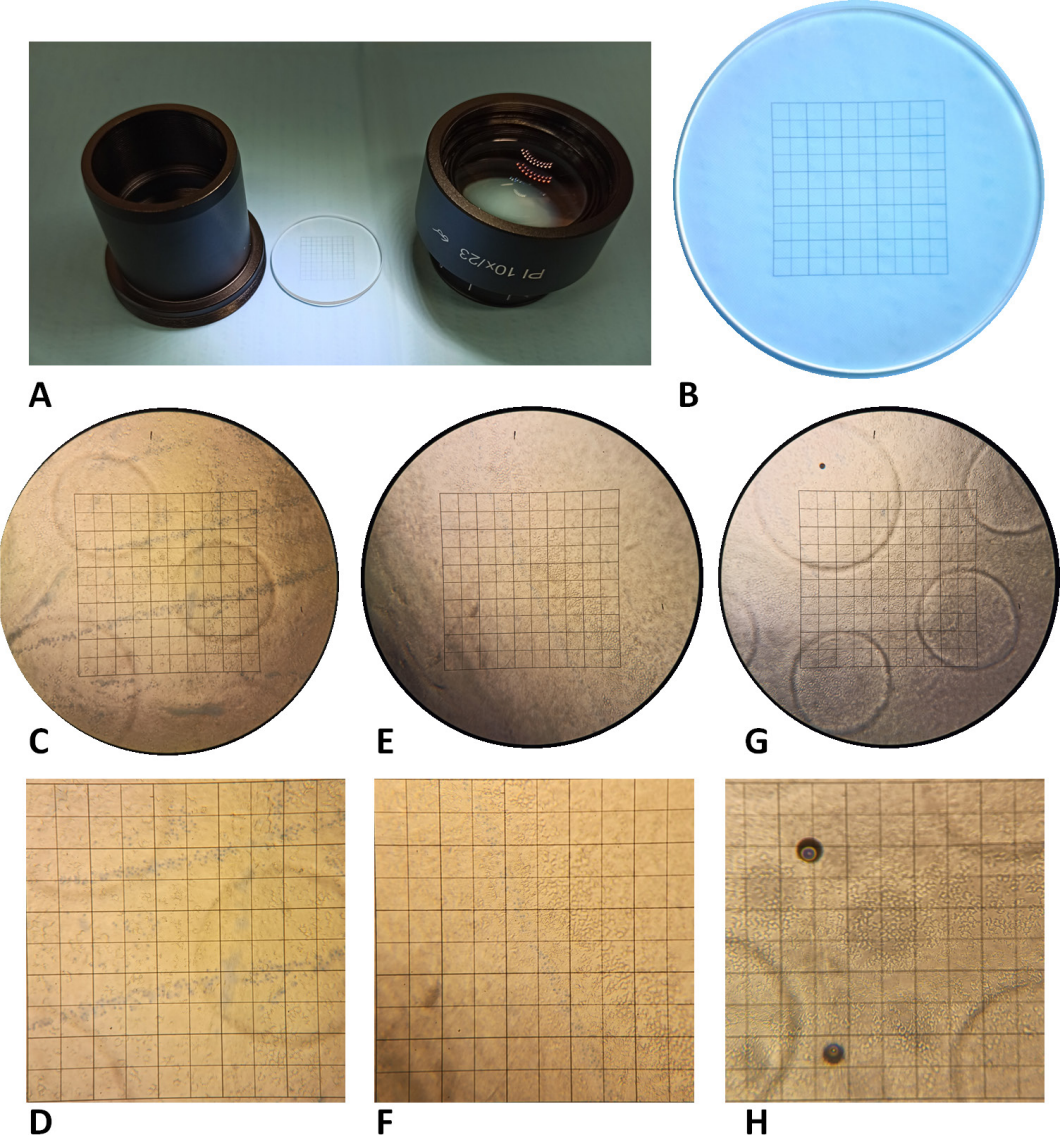
The set-up used for tissue analysis. (A) Eyepiece with the (B) reticule grid used for counting the endothelial cell density (ECD) and trypan blue positive cells (TBPCs). ECD and TBPCs as observed subsequently after (C–D) graft 1, (E–F) graft 2 and (G–H) graft 3.

The tissues were washed in sterile phosphate buffered saline (PBS-1X) and the endothelial cells were stained with trypan blue (0.25%, VisionBlue, DORC, Zuidland, Netherlands) for 30 s. After washing, the tissues were placed in a 35 mm Petri dish prefilled with a hypotonic sucrose solution with the epithelial side facing the top. Corneal endothelial cell mortality (trypan blue positive cells (TBPC)) and density (cells/mm^2^—reticule count) (figure 1A,B) were manually counted by a single senior researcher to avoid counting errors using an inverted light microscope (AxioVision, Zeiss, Oberkochen, Germany) before and after stripping (figure 1C–H).^16^

### At the wet lab

The wet lab was initiated with a talk and videos explaining the details of the surgical manoeuvres. Eight trainee surgeons were paired up and asked to record the time, error, discard rate and unsuccessful preparation errors of their respective partners. The technique used at the wet lab was stripping by scoring the peripheral endothelium.^17^ Briefly, the trainee surgeons placed the donor cornea with the endothelial side up on the corneal punch block. Using a Sinskey hook, the trainee surgeons created a partial break on the peripheral corneal endothelium, about 1 mm from the trabecular meshwork. After a quick wash with PBS, trypan blue dye was applied for 30 s to stain the cut edge, and then the cornea was rinsed again with PBS to remove the excess trypan blue. Descemet membrane (DM) cleavage hook was used to separate the peripheral cut edge of DM from the underlying stroma throughout the 360-degree circumference. A few drops of PBS were applied to the endothelium to avoid drying. Using the tying forceps, the free edge of the DM was gently grasped, and the peeling was performed towards the opposite end from the point of initiation. The peeling was performed using a single quadrant method or in different quadrants depending on the adhesive property of the tissue. The DM was peeled leaving a peripheral hinge, or if peeled completely then placed in PBS.

### Data collection and statistical analysis

The tissues were stained with trypan blue, washed with PBS and placed in sucrose solution to visualise the endothelial cell borders. The overall cell density and the TBPC were counted using an in-built reticule of a light microscope on-site. The endothelial cell density (ECD), mortality, time, peripheral and central tears and complete peel-off were recorded and analysed.

### Statistical analysis

Descriptive data were summarised using the mean (SD), median (IQR) and number (percentages) as appropriate. Repeated measures analysis of variance tests were used to evaluate the learning effect of the three consecutive trials on the main effects of time on ECL, percentage of TBPCs, time to complete the peel, prevalence of peripheral and central tears and complete peel-off. Post hoc pairwise t-tests with Bonferroni correction for multiple comparisons were then used for comparisons between each trial. The data were also confirmed by analysing the later grafts with the first graft using Friedman repeated measures non-parametric test with post hoc Dunn’s test.

To verify the effect of peel time on ECL and mortality, linear mixed model (LMM) with time to complete the peel as the fixed effect was employed, and random intercept and random slope over time for each subject as a random effect.

A p value of 0.05 (95% CI) was considered statistically significant. All analyses were conducted using R software V.4.2.2 (R Project for Statistical Computing, Vienna, Austria).

## Results

**Figure 2:**
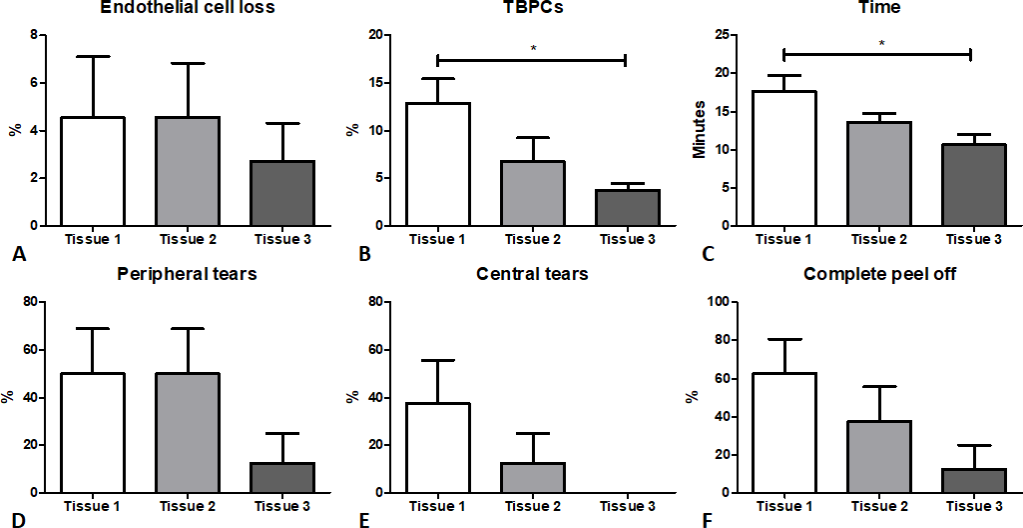
Statistical analysis using one-way analysis of variance (ANOVA) to compare the cumulative difference between grafts 1–3. (A) Endothelial cell loss (%), (B) trypan blue positive cells (TBPC, %), (C) time taken to peel the grafts (minutes), (D) peripheral (%) and (E) central tears (%), and (F) complete peeling off the graft (%). The data are represented as mean±SEM. *P<0.05.

**Figure 3:**
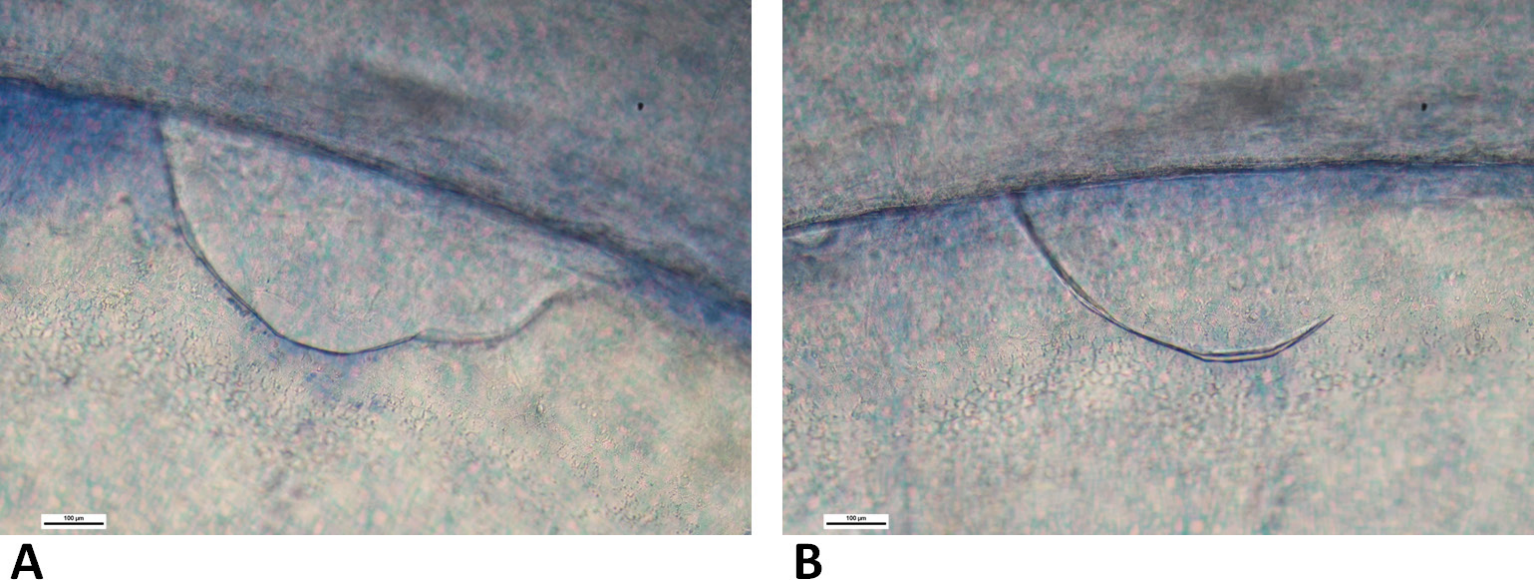
Tears observed during the learning curve. (A) Peripheral tear after cutting the endothelium due to improper handling and (B) hairline tear leading to a possible larger tear, which was rescued by initiating the peel from the opposite end and using the tear as a hinge.

Eight corneal trainee surgeons participated in the wet lab. Each participant stripped three DMEK grafts for a total of 24 tissues. The summary data of the experiment are reported in table 1. The mean (SD) ECD (cells/mm^2^) recorded was 1638 (212), 1663 (166) and 1606 (159) before preparation, which decreased to 1556 (182), 1588 (162) and 1560 (145) after preparation from tissues 1, 2 and 3, respectively. A learning effect was observed for the time to complete the peeling of the DMEK graft, as the average time decreased with consecutive trials. Although the ECL did not differ between the first and the last tissue (mean (SD), 4.5% (7.3%) vs 2.7% (4.5%), p>0.05; figure 2A), a significant difference in mortality of the tissue expressed as the percentage of TBPC (mean (SD), 12.9% (7.2%) vs 3.8% (2%), p=0.0489; figure 2B) as well as time to complete the peel between the first and last tissues was observed (mean (SD), 17.6 (6.1) vs 10.6 (3.9) min, p=0.0469; figure 2C). Although not significant, a general decreasing trend was observed between the first and the last trial in creating peripheral tears (mean (SD), 50% (53.5%) vs 12.5% (35.4%), p>0.05; figure 2D), central tears (mean (SD), 37.5% (51.7%) vs 0% (0%), p>0.05; figure 2E) and percentage of complete peels (mean (SD), 62.5% (51.8%) vs 12.5% (35.4%), p>0.05; figure 2F). The peripheral tears during the first attempts lead to either DM chip-off (figure 3A) or a hairline tear (figure 3B) leading to a larger DM tear.

**Table 1:** Descriptive statistics obtained from tissues 1, 2 and 3 performed by eight trainee corneal surgeons

	Tissue 1	Tissue 2	Tissue 3	rmFriedman
Time (min), mean (SD)	17.6 (6.1)	13.6 (3.1)	10.6 (3.9)	**0.0469**
ECL (%), mean (SD)	4.5 (7.3)	4.6 (6.4)	2.7 (4.5)	0.7943
TBPCs (%), mean (SD)	12.9 (7.2)	6.8 (7.1)	3.8 (2)	**0.0489**
Central tears (%), mean (SD)	38 (51.8)	13 (35.4)	0 (0)	0.1495
Peripheral tears (%), mean (SD)	50 (53.4)	50 (53.4)	12.5 (35.4)	0.2851
Complete peel (%), mean (SD)	63 (51.8)	38 (51.8)	13 (35.4)	0.1495

Bold values represent statistical significance (p<0.05).

ECL, endothelial cell loss; rmFriedman, repeated measures non-parametric Friedman; TBPCs, trypan blue positive cells.

**Figure 4:**
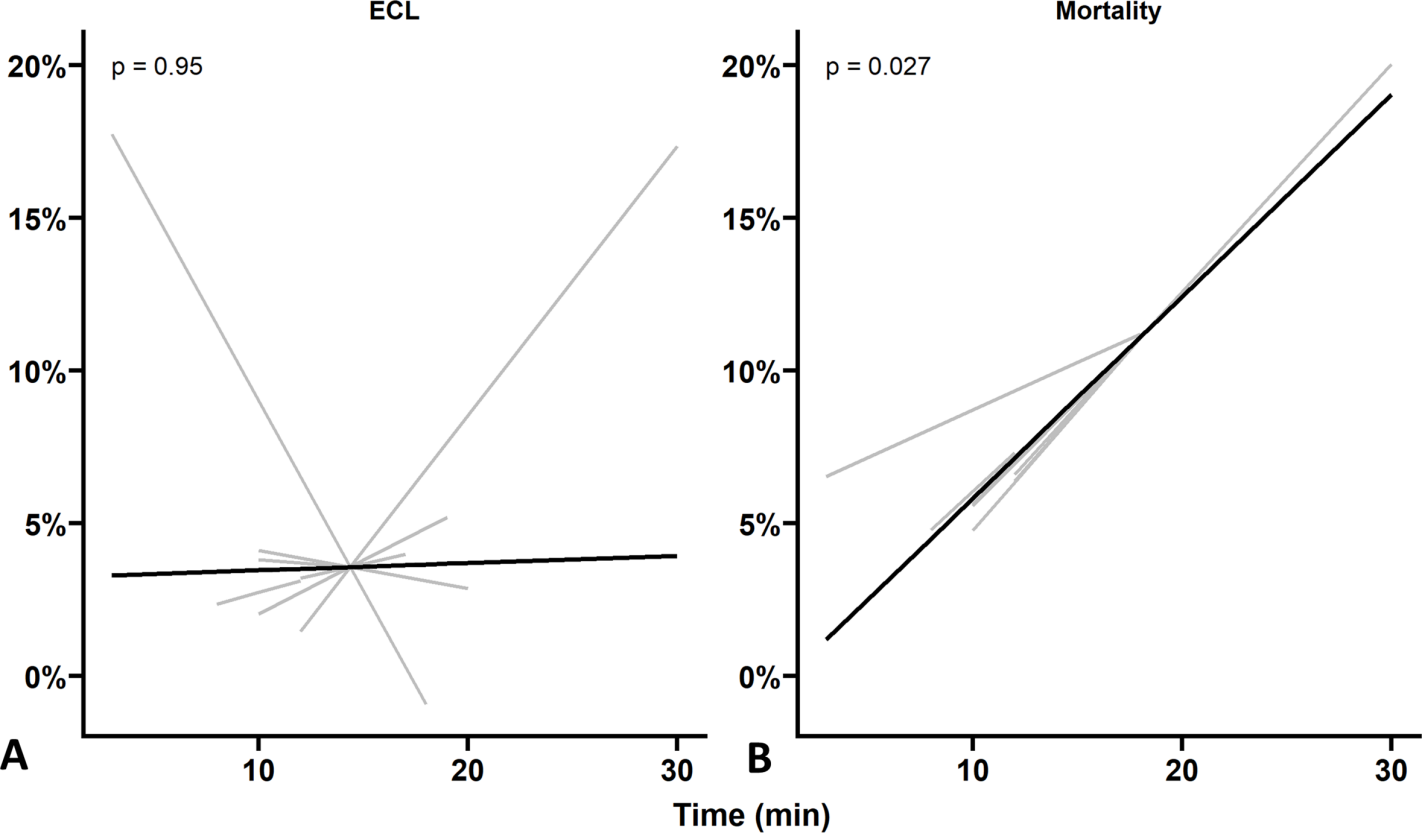
Linear mixed model analysis. A linear relationship between peeling time and (A) endothelial cell loss (ECL) and (B) trypan blue positive cells (TBPCs). The solid black lines represent the population level fit, while the grey lines represent the individual fellow (n=8) level fit.

Generalised LMMs showed no correlation of time to complete the peel with ECL (% (SE), 0.023 (0.386), p=0.74; figure 4A). Instead, a correlation between the time to complete the peel and TBPCs was found, as longer peeling times were associated with higher endothelial mortality values (% (SE), 0.718 (0.225), p=0.001; figure 4B). This meant that for each additional minute of peeling time, there was an approximate 0.7% increase in the TBPC proportion expected.

## Discussion

This study investigated and reported the performance of trainee surgeons in a DMEK wet lab using ex vivo human corneas. Our objective was to analyse the performance outcomes to further the understanding of the role of wet labs in enhancing the training of DMEK graft preparation.

DMEK surgery has emerged as the superior corneal transplant for endothelial dysfunction, surpassing PK and DSAEK owing to the minimal ECL, rapid visual recovery, improved visual outcomes and a lower rejection rate.^1–5^ Effective DMEK training methods are required to increase the wider adoption and transition to DMEK on account of the steep learning curve and technical ability required.^6^

Wet labs play a crucial role as a risk-free DMEK learning tool taking place in a controlled setting, free from complications or failure.^12 18^ DMEK surgical steps are simulated through wet labs across a variety of settings, which through the years have been improved to better emulate the dynamics of real-life surgery.^13 19–22^ A survey conducted identified that participation in DMEK wet labs ranks among the most helpful learning experiences leading up to a trainee surgeon’s first DMEK cases.^9^

The aim of wet labs stretches beyond merely familiarising trainees with the preparation to surgical steps. Wet labs strive to instil a deep understanding of the anterior and posterior segment pressure dynamics, which govern the flow of the surgery, as well as to standardise the procedure as much as possible, leaving minimal individual improvisation or chance.^19 20^

The proposed benefits of wet labs encompass improved surgeon ability, microsurgical skills and DMEK success rates.^12 23^ Our findings indicate a significantly reduced mortality of endothelial cells (TBPC) between the first and last attempts (p=0.01), serving as evidence for these benefits. Ultimately, a greater number of endothelial cells transplanted translate to enhanced graft performance and patient outcomes.^24^

DMEK graft preparation’s steep learning curve has profoundly hindered its widespread adoption.^6^ Our data reveal that trainee surgeons become significantly quicker in performing the DMEK peel by their last attempt compared with their first. The significant correlation found between longer peeling times and higher TBPC as well as the approximate increase of 0.7% mortality for each additional minute required to complete the peel may also explain the learning effect. This demonstrates the unique possibility of wet labs to aid trainee surgeons in overcoming the learning curve in a safe and controlled environment.

DMEK surgery is highly technical and precise.^12 25^ Trainee surgeons may struggle to execute certain intricate surgical steps including the peeling of the graft leaving a hinge.^26^ The percentage of complete peels (not leaving the hinge) in our study significantly reduced from the initial to the last attempt. Although peripheral breaks and tears were observed during the first attempts (figure 3), our study showed a diminishing percentage of central and peripheral graft tears with each successive attempt. This suggests that wet labs can play a role in developing trainee surgeons’ confidence and competence in performing DMEK surgery.

Unfortunately, the wider expansion of human cornea DMEK wet labs has been limited by several factors. Although the cost to attend is high, the limited availability of corneas for training remains the greatest barrier. These challenges become particularly pronounced when compared with wet labs using animal tissue or vegetable matter.^12^ Wet labs are also not widely accessible leaving some trainee surgeons without this valuable hands-on experience.^12^ Global situations such as pandemics can negatively affect in-person training, wet labs could struggle to withstand such threats leading to long-term impacts on trainee surgeons.^18 27 28^ Lastly, at present, wet labs only impart basic skills and are not equipped to teach complex cases.^12^ However, solutions to some of these limitations are possible. Forming partnerships with neighbouring institutes and professional societies (eg, European Eye Bank Association and Eye Bank Association of America) can reduce expenses through shared space and skills as well as enhance the curriculum to include more complex cases.^12^ Continual performance data collection can identify training gaps and remote wet labs can overcome accessibility issues.

This study is limited by trainee surgeons only having three DMEK attempts and did not contain a control graft without manipulation. In addition, the sample size was small, possibly affecting the precision of data modelling, as estimates of variance of random effects by LMMs in case of small sample sizes may prove to be unstable and models can be affected by overfitting. While challenging to achieve, incorporating either more attempts per trainee surgeon or involving a larger number of trainee surgeons would provide further evidence of the surgical skill improvement obtained through wet labs. Multicentre wet lab studies may be considered though differing variables could make data analysis difficult. Additionally, we recommend performance evaluation during wet labs to identify key factors that are limiting the uptake of DMEK, both preparation and delivery.

## Conclusion

DMEK wet labs using ex vivo human corneas provide a vital hands-on learning opportunity, fostering confidence, technical ability and procedural standardisation in a risk-free setting. Our study highlights the significant improvement in DMEK peel completion time, the number of endothelial cells transplanted and successful graft peels. These findings help validate the benefits of human cornea DMEK wet labs and their pivotal role in enhancing medical education and patient outcomes. This emphasises the importance of continued support for the expansion of this valuable learning resource, promoting wider uptake of DMEK surgery, and sets the stage for evaluating the performance outcomes of the trainees in the wet labs.

## Data Availability

No data are available.
